# Seed Density as a New Predictive Index of Seed Migration in Brachytherapy for Prostate Cancer Using Iodine-125 Loose Seed

**DOI:** 10.3390/curroncol30040308

**Published:** 2023-04-04

**Authors:** Takahiro Yamaguchi, Masayuki Matsuo, Takayuki Mori, Yoshifumi Noda, Chiyoko Makita, Fuminori Hyodo, Koji Iinuma, Masahiro Nakano, Takuya Koie, Hidekazu Tanaka

**Affiliations:** 1 Department of Radiology, Gifu Municipal Hospital, Gifu 5008513, Japan; 2 Department of Radiology, Gifu University Graduate School of Medicine, Gifu 5011194, Japan; 3 Gifu University Institute for Advanced Study, Gifu 5011193, Japan; 4 Department of Urology, Gifu University Graduate School of Medicine, Gifu 5011194, Japan; 5 Department of Urology, Gifu Prefectural General Medical Center, Gifu 5008717, Japan; 6 Department of Radiology, Yamaguchi University Graduate School of Medicine, Ube 7558505, Japan

**Keywords:** prostate cancer, brachytherapy, seed migration, low dose rate, Iodine-125

## Abstract

Aim: This study aimed to examine the usefulness of seed density as a predictor of seed migration in patients with prostate cancer who received brachytherapy using Iodine-125 loose seed. Methods: From May 2006 to April 2016, 320 patients with localized prostate cancer underwent transperineal brachytherapy using iodine-125 loose seeds. Among them, 202 (63.1%) patients received brachytherapy monotherapy and 118 (36.9%) received combined brachytherapy and external beam radiotherapy. Seed density was calculated using the following formula: seed density = implanted seed number/prostate volume. All patients underwent radiography of the chest, abdomen and pelvis, and computed tomography at 1 day, 1 month, and 1 year after brachytherapy to evaluate the presence of seed migration. Results: In total, the number of implanted seeds was 21,876. Seed migration was detected in 92 (28.8%) patients. Of a total of 21,876 seeds, 144 (0.66%) showed migration. The number of needles, number of seeds, and seed density were significantly higher in the group with migration than in the group without migration (*p* = 0.05). The ROC cutoff values for prostate volume, number of needles, number of seeds, and seed density were 20.9 cc, 21, 65, and 3.0, respectively. In the univariate analysis, prostate volume, number of needles, number of seeds, seed density, and treatment modality were all significant factors in predicting migration (*p* = 0.05). In the multivariate analysis, seed density and treatment modality were significant factors in predicting migration (*p* = 0.05). Conclusion: Seed density is useful for predicting seed migration. In cases with seed density > 3.0, it is necessary to take measures such as considering the use of stranded seeds.

## 1. Introduction

Prostate cancer is the second most common male cancer worldwide, with a global estimated number of new cases in 2020 of 1,414,259. In contrast, in the number of cancer-related deaths, prostrate cancer was ranked fifth among males [1]. Patients with localized prostate cancer have many treatment options, such as surgery, radiotherapy, and androgen deprivation therapy [2]. Radiotherapy includes intensity-modulated radiotherapy, stereotactic body radiotherapy, particle therapy, and low-dose rate and high-dose rate brachytherapy [3,4,5,6,7,8]. Low-dose rate brachytherapy with iodine-125 has been demonstrated to be an effective and safe treatment [2,7,9].

The migration rate of loose seeds was reported to be higher than that of stranded seeds [10,11]. However, a high degree of freedom in placement is one of the benefits of loose seeds.

Implanted seed numbers and inserted needle numbers have been reported as predictors of migration in brachytherapy for prostate cancer using loose seeds [12,13,14].

It is thought that the insertion of many needles or the placement of many seeds in the limited volume of the prostate facilitates migration. However, even with the same number of seed placements, the possibility of migration is expected to differ among cases with large and small prostate volumes. Therefore, we devised an index, a seed density, that considers not only the number of implanted seeds but also the prostate volume.

This study aimed to examine the usefulness of seed density as a predictor of migration.

## 2. Materials and Methods

The present study was approved by the Human Research Committee of the Institutional Review Board of our hospital and complied with the guidelines of the Health Insurance Portability and Accountability Act of 1996 and the Declaration of Helsinki. Due to the retrospective nature of the study, the requirement for informed consent was waived.

### 2.1. Patients

From May 2006 to April 2016, 320 patients with localized prostate cancer underwent transperineal brachytherapy using iodine-125 loose seeds. Patient characteristics are shown in Table 1. The median patient age was 66 years (range, 50–78 years). The median PSA level at pretreatment was 6.3 ng/mL (range, 1.7–55.0 ng/mL). A total of 187 (58.4%), 100 (31.3%), 11 (3.4%), 19 (5.9%), and 3 (0.9%) patients had cT1c, T2a, T2b, T2c, and T3a, respectively. The patients had a Gleason Score (GS) of ≤6 (*n* = 151 [47.2%]), 7 (*n* = 144 [45.0%]), 8 (*n* = 18 [5.6%]), and 9 (*n* = 7 [2.2%]). Among them, 202 (63.1%) patients received brachytherapy monotherapy and 118 (36.9%) received combined brachytherapy and external beam radiotherapy. The radioactive source strength varied from 0.31 to 0.38 mCi per seed.

### 2.2. Pre-Plan

One month before the procedure, computed tomography (CT) and magnetic resonance imaging (MRI) of the pelvis were performed as a pre-plan to simulate implantation. An order was placed for the number of seeds used in the pre-plan, plus a few extra seeds. Treatment was planned using a peripheral approach. The prescribed doses were 145 Gy and 114 Gy for monotherapy and combined therapy, respectively. VariSeed (Varian Medical Systems, Palo Alto, CA, USA) was used both in calculating the preoperative prostate volume and in planning seed placement. Extraprostatic seed placement was not planned.

### 2.3. Brachytherapy

Treatment was performed using standard techniques. All patients underwent lumbar spinal anesthesia. Using transrectal ultrasound as a guide, the needles were inserted transperineally into the peripheral position of the prostate and peripheral seeds were implanted. Next, the needles were placed inside the prostate and internal seeds were also placed.

### 2.4. Evaluation of Migration

All patients underwent radiography (orthogonal chest radiographs, an abdominal radiograph, and a posteroanterior pelvic radiograph) and CT at 1 day, 1 month, and 1 year after brachytherapy to evaluate the presence of seed migration. In this study, seed migration was defined as an event in which seeds enter a blood vessel and are transported out of the prostate by blood flow. Hence, the cases in which the seeds moved to the periprostatic tissue were not counted as migration. In other words, the cases in which the seeds were detected radiographically out of the anatomical location were counted as migration.

### 2.5. Statistics

Seed density was calculated using the following formula: seed density = implanted seed number / prostate volume. The Mann–Whitney U test was used to compare the variables between the groups with and without migration.

A *p* value < 0.05 was considered statistically significant.

Cut-off values for predicting the occurrence of migration were determined using receiver operating characteristic (ROC) for prostate volume, number of needles, number of seeds, and seed density. These variables were divided into two groups based on the cut-off values determined by ROC. Univariate and multivariate logistic regression analyses were performed to predict the occurrence of migration using these variables.

## 3. Results

In total, the number of implanted seeds was 21,876. The median prostate volume was 24.5 cc (range, 11.3–54.5). The median number of needles inserted was 22 (range, 13–35). The median number of seeds per patient was 66 (range, 38–108).

**Figure 1:**
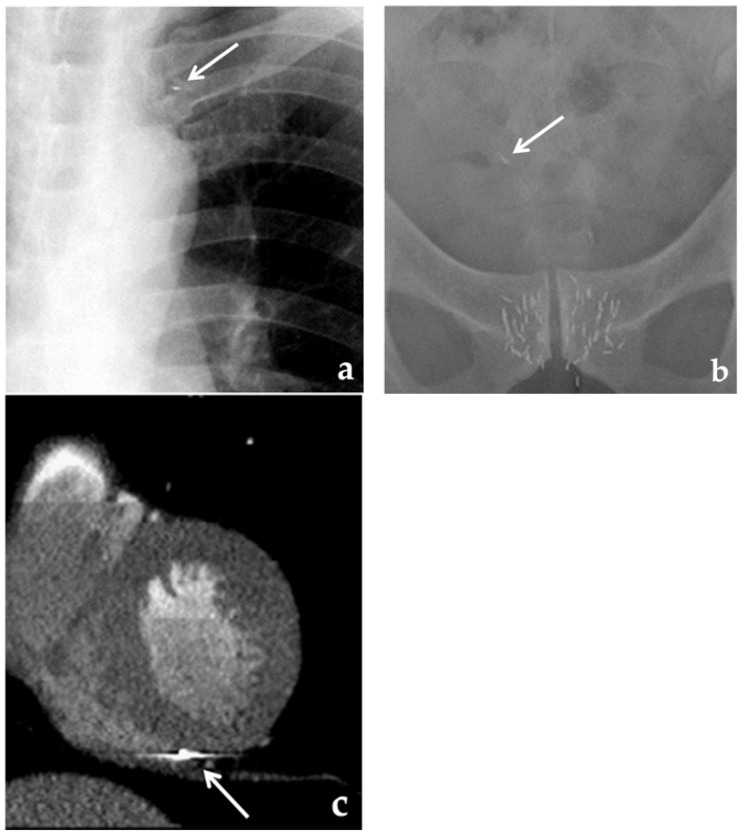
Seed migration after brachytherapy. A follow-up orthogonal chest radiograph showed that a seed had migrated to the left upper lung (solid arrow) (**a**). A follow-up posteroanterior pelvic radiograph showed that a seed had migrated to the intra pelvic area (solid arrow) (**b**). Contrast-enhanced CT showed that the seed had migrated to the right ventricle (solid arrow) (**c**).

Seed migration was detected in 58 patients (18.1%) 1 day after brachytherapy. One month later, 31 additional cases showed migration for a total of 89 cases (27.8%). One year later, 3 additional cases of migration were observed for a total of 92 cases (28.8%). At 1 year, the location of migrated seeds in the pelvis occurred in 89 cases (27.8%), in the lung in 47 cases (14.7%), and others in 8 cases (2.5%). Others included two kidneys, one right atrium, one spinal canal, one left diaphragm, one peritoneum, one right thigh, and one unknown site (Figure 1).

Of a total of 21,876 seeds, 144 (0.66%) showed migration.

Table 2 summarizes the median number of variables of patients stratified by the results of seed migration. The number of needles, number of seeds, and seed density were significantly higher in the group with migration than in the group without migration (*p* = 0.000324, 0.0000351, and 0.0142, respectively). The participants in the group with migration tended to have smaller prostate volumes than those in the group without migration (*p* = 0.0651).

The results of univariate and multivariate analysis are summarized in Table 3. The ROC cutoff values (AUC, sensitivity, and specificity) for prostate volume, number of needles, number of seeds, and seed density were 20.9 cc (0.566, 0.772, 0.368), 21 (0.631, 0.707, 0.504), 65 (0.648, 0.739, 0.535), and 3.0 (0.588, 0.402, 0.772), respectively. In the univariate analysis, prostate volume, number of needles, number of seeds, seed density, and treatment modality were all significant factors in predicting migration (*p* = 0.0167, 0.000705, 0.0000139, 0.00342 and 0.0000038, respectively). In the multivariate analysis, seed density (*p* = 0.0232) and treatment modality (*p* = 0.028) were significant factors in predicting migration.

## 4. Discussion

Seed migration may occasionally be observed during brachytherapy with loose seeds. Migration was reported to be observed in 22.2–36.2% [12,13,14]. Migration to the vertebral venous plexus has also been reported in rare cases [15]. Our data also showed migration in 28.6% of the samples, which is comparable to findings in previous reports. In recent years, stranded seeds with a low risk of migration have also become available.

However, loose seeds, which have a high degree of freedom in terms of seed placement, are still used. In brachytherapy using iodine-125 for prostate cancer, a large number of seeds are conventionally placed; therefore, even if a small number of seeds migrate, the negative effect on the dose distribution is small [16,17]. Migration is often observed in the lungs; however, migrated seeds rarely cause adverse events [18]. However, on rare occasions, migrated seeds can cause symptomatic adverse events, such as radiation pneumonitis and myocardial infarction [19,20]. 

It has been reported that migration is significantly more likely to occur when there are many inserted needles and implanted seeds [12,13,14]. Intuitively, it is easy to understand that when many seeds are placed in the limited space of the prostate, some of the seeds do not remain in the prostate and are prone to migration. It has been reported that extraprostatic placement is a risk factor for migration [21]; however, extraprostatic placement is inevitable with excessive seed placement. However, these risk factors do not include host-side characteristics. Even when the same number of seeds is implanted, seed placement is more difficult in cases where the prostate gland has a smaller volume than in a larger one. Similarly, it is naturally more difficult to place a large number of seeds than a small number of seeds in cases with the same prostate volume. Monotherapy usually requires more seeds than combined therapy, which makes seed implantation more difficult. We devised seed density as an index that considers both the number of seed placements and prostate volume. This measures the number of seeds per unit volume of the prostate. In the ROC analysis, the cutoff value of seed density for predicting the presence or absence of migration was 3.0. Univariate analysis revealed that prostate volume, number of needles, number of seeds, seed density, and treatment modality were all significant factors. However, only seed density and treatment modality remained significant factors in the multivariate analysis. Seed density is considered to be more useful than previously reported predictors of migration, such as the numbers of seeds and needles. In cases with a seed density > 3, it is necessary to take measures such as considering the use of stranded seeds.

## 5. Conclusions

We examined the usefulness of seed density as a new predictor of migration after brachytherapy that considers both the number of seed placements and prostate volume. In the ROC analysis, the cutoff value of seed density for predicting the presence or absence of migration was 3.0. Univariate analysis revealed that prostate volume, number of needles, number of seeds, seed density, and treatment modality were all significant factors. However, only seed density and treatment modality remained significant factors in the multivariate analysis. The seed density that we formulated might help predict seed migration and help develop treatment strategies.

## Figures and Tables

**Table 1:** Patient characteristics (*N* = 320).

Characteristic	Patients (%)	Median (Range)
Age (years)		66 (50–78)
Tumor stage		
T1c	187 (58.4)	
T2a	100 (31.3)	
T2b	11 (3.4)	
T2c	19 (5.9)	
T3a	3 (0.9)	
Pretreatment PSA (ng/mL)		6.3 (1.7–55.0)
<4.0	11 (3.4)	
≥4.0, <10.0	257 (80.3)	
≥10.0	52 (16.3)	
Gleason score		
3 + 3	151 (47.2)	
3 + 4	105 (32.8)	
4 + 3	39 (12.2)	
3 + 5	2 (0.6)	
4 + 4	16 (5.0)	
4 + 5	6 (1.9)	
5 + 4	1 (0.3)	
Prostate volume (cc)		24.5 (11.3–54.5)
Needles inserted		22 (13–35)
Seeds implanted		66 (38–108)
Seed density		2.7 (1.5–4.8)
Treatment modality		
Monotherapy	202 (63.1)	
Combined therapy	118 (36.9)	

**Table 2:** Median number of variables of patients stratified by results of seed migration.

	Seed Migration (+) (*n* = 92)	Seed Migration (-) (*n* = 228)	*p* Value
Prostate volume (cc)	26.2	24.2	0.0651
Needles	22	20	0.000324 *
Seeds	75	63	0.0000351 *
Seed density	2.78289	2.666311	0.0142*

* Significant difference in values was found between the group with migration and the group without migration (*p* < 0.05).

**Table 3:** Results of univariate and multivariate analysis.

		Univariate Analyses			Multivariate Analyses		
		Odds Ratio	95% CI	*p* Value	Odds Ratio	95% CI	*p* Value
Prostate volume (cc)	20.9< vs. ≥20.9	1.97	1.13–3.44	0.0167 *	1.33	0.55–3.22	0.531
Needles inserted	≥21 vs. 21<	2.45	1.46–4.11	0.000705 *	1.47	0.76–2.86	0.256
Seeds implanted	≥65 vs. 65<	3.26	1.91–5.56	0.0000139 *	1.56	0.67–3.67	0.306
Seed density	≥3.0 vs. 3.0<	2.18	1.29–3.66	0.00342 *	2.10	1.11–3.98	0.0232 †
Treatment modality	Mono vs. combined	4.23	2.29–7.80	0.0000038 *	2.28	1.09–4.76	0.028 †

Note.—95% CI: 95% confidence interval. * Significant factors for predicting migration in the univariate analysis (*p* < 0.05). † Significant factors for predicting migration in the multivariate analysis (*p* < 0.05).

## Data Availability

The data presented in this study are available on request from the corresponding author. The data are not publicly available due to privacy and ethical reasons.
